# Impella to Treat Acute Myocardial Infarct-Related Cardiogenic Shock

**DOI:** 10.3390/jcm11092427

**Published:** 2022-04-26

**Authors:** Jacob Eifer Møller, Jesper Kjaergaard, Christian Juhl Terkelsen, Christian Hassager

**Affiliations:** 1 Department of Cardiology, Odense University Hospital, 5000 Odense, Denmark; 2 Heart Center, Copenhagen University Hospital Rigshospitalet, 2100 Copenhagen, Denmark; jesper.kjaergaard.05@regionh.dk (J.K.); christian.hassager@regionh.dk (C.H.); 3 Department of Cardiology, Aarhus University Hospital, 8000 Aarhus, Denmark; christian.terkelsen@skejby.rm.dk

**Keywords:** cardiogenic shock, acute myocardial infarction, mechanical circulatory support

## Abstract

Acute myocardial infarction complicated by cardiogenic shock (AMICS), is characterized by critically low cardiac output and decreased myocardial contractility. In this situation, a treatment that unloads the myocardium and restores CO without increasing the myocardial oxygen demand is theoretically appealing. Axial flow pumps offer hemodynamic support without increasing myocardial oxygen consumption. Consequently, the use of axial flow pumps, especially the Impella devices, is increasing. It is likely that the SCAI C patient with predominantly left ventricular failure and without prolonged cardiac arrest is the best candidate for these devices. Registry data suggest that pre-PCI Impella may be advantageous to post-PCI placement. However, several gaps in knowledge exist regarding optimal patient selection, futility criteria, timing, weaning and escalation strategy, and until data from adequately sized randomized trials are available, immediate individual evaluation for mechanical circulatory support by a shock team is warranted when a patient is diagnosed with AMICS.

## 1. Introduction

Acute myocardial infarction (AMI), complicated by cardiogenic shock (AMICS), occurs in 5–10% of AMI cases. Even with immediate revascularization, pharmacological, and in selected cases, mechanical circulatory support, mortality often approaches 50% [1,2]. Classically, the condition is a consequence of acute myocardial dysfunction with critically reduced cardiac output and subsequent organ hypoperfusion and organ failure. Active ventricular unloading with transvalvular axial flow devices seems promising for the restoration of CO without increasing myocardial oxygen consumption [3,4]. Evidence to guide this treatment is, however, largely based on expert consensus, conflicting retrospective studies, and small underpowered randomized trials. Thus, several gaps in evidence exist, including optimal patient identification, the timing of treatment, futility criteria, the duration of treatment as well as weaning and escalation strategies.

## 2. Axial Flow Pump Technology

Impella (Abiomed, Danvers, MA, USA) is by far, the most frequently used transvalvular flow device, with options for mechanical circulatory support both for the left and right ventricle. Left-sided devices include the Impella 2.5 and CP with 13 or 14F percutaneous arterial access, and the Impella 5.0 and 5.5 devices that need surgical access. The Impella RP is a right-sided device that uses a percutaneous 22F venous access. These devices generate continuous flow. In AMICS, the Impella CP is often preferred in the acute setting, thus providing a continuous flow of up to 3.8 L/min [5]. 

## 3. Pathophysiological Rationale for Axial Flow Pump in AMICS

After coronary artery occlusion, contractility will decrease and eventually cease in the affected myocardial territory unless there are adequate collaterals. Even with the restoration of coronary flow, the recovery of contractility often takes several hours and continues to improve over days [6]. Transvalvular left-sided axial flow pumps aspirate blood from the left ventricle (LV) and eject it into the ascending aorta which will cause ventricular unloading with decreased wall stress, reduced myocardial oxygen consumption and improvement in cardiac output, leading to an increase in coronary and systemic perfusion [4,7,8]. As opposed to centrifugal flow pump technology, which is used in veno-arterial extracorporeal membrane oxygenation (VA-ECMO), axial flow pumps augment mostly diastolic blood pressure and may reduce systolic blood pressure with a modest effect on the mean arterial blood pressure [9]. 

## 4. Patient Identification

Careful patient selection is a key factor in achieving acceptable outcomes in patients treated with axial flow pumps or other MCS systems. These complex invasive treatments are costly and with potentially serious complications. Thus, rigorous patient selection criteria are pivotal. AMICS is a heterogeneous condition that may be seen in ST-segment elevation AMIs as well as non-ST-segment elevation AMIs, and frequently, it is preceded by cardiac arrest [10]. Although most frequently associated with predominantly LV failure, AMICS can also be seen with predominantly right ventricular (RV) or biventricular failure, or due to mechanical complications. Thus, several phenotypes of AMICS exist that likely should not be managed in the same way. 

**Figure 1:**
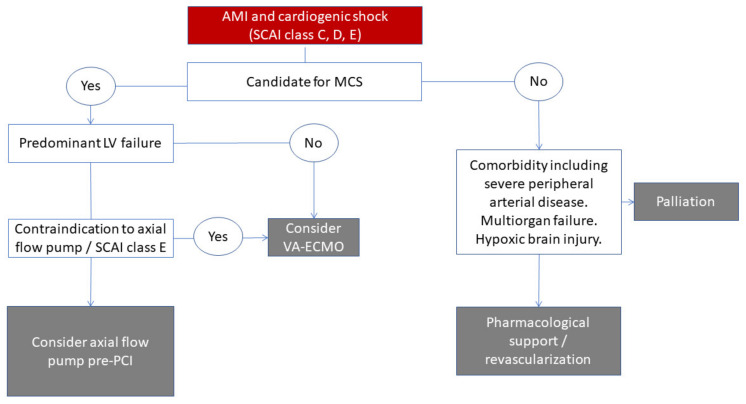
Flow chart on the decision process in patient selection for mechanical circulatory support among patients with acute myocardial infarct-related cardiogenic shock, see text for details. MCS, mechanical circulatory support; SCAI, Society for Cardiovascular Angiography and Interventions; VA-ECMO, veno-arterial extracorporeal membrane oxygenation.

AMICS is also a spectrum of severity of disease that recently has been categorized into five classes by the Society for Cardiovascular Angiography and Interventions (SCAI) [11]. SCAI class C includes those with manifest hypoperfusion, as opposed to those at risk of developing CS (SCAI class A and B). With a continuous flow of up to 3.8 L/min, the Impella CP provides a significant flow but usually not a full restoration of flow, and the pump requires adequate RV function or low pulmonary vascular resistance. Thus, the best candidate for this device would be the hypoperfused SCAI class C patient, with some intrinsic LV function and sufficient RV function able to provide adequate LV filling. The SCAI D (deteriorating) patient will likely in some cases benefit from axial flow pumps, however, in the most severe cases and in SCAI E (extremis), patients may be better suited for VA-ECMO. Therefore, in patients evaluated for MCS, it is mandatory to perform an immediate evaluation of cardiac function and identification of potential conditions that would preclude the use of axial flow devices, such as LV thrombus, moderate/severe aorta valve regurgitation, and a mechanical complication to AMI. For this purpose, echocardiography is especially well suited to providing immediate real-time images that also can be used to guide the placement of the device, as demonstrated in Figure 1. Invasive hemodynamics using right heart catheterization provides important hemodynamic insight, although this will not provide direct insight into valvular function, LV thrombus, or regional wall motion, and this approach is more time-consuming in a situation where the time to opening the infarct-related coronary artery is pivotal. Finally, hypoperfusion should be confirmed, either metabolically using an assessment of elevated blood lactate >2 mol/L [12] or, if not possible, by using classic clinical criteria (cold clammy skin, altered mental status, oliguria), although these may be unspecific and subjective.

About 40% of AMICS patients will present with out-of-hospital cardiac arrest (OHCA) [1,10]. Despite similar hemometabolic characteristics on admission, the underlying pathophysiology associated with whole-body ischemia due to the abrupt cessation of circulation and subsequent post cardiac arrest syndrome will have a different course than the hypoperfused patient without cardiac arrest [13]. Thus, special attention should be paid to the identification of patients with a need for MCS in the OHCA population, based on an immediate response to volume optimization, vasopressors and repeated echocardiographic evaluation.

## 5. Futility Criteria

Some patients with AMICS will die regardless of MCS and immediate revascularization, and several risk models have been proposed for patients with cardiogenic shock, however, with moderate accuracy [14,15]. Most of these models have been developed in populations where the majority of patients were not treated with MCS, and the same risk factors may not apply in those selected for MCS [16]. Advanced age alone is a controversial futility criterion, and acceptable outcomes have been reported for Impella treatment in the elderly [17]. However, data on patients more than 80 years of age is very limited and evidence for use of Impella in this patient group is practically nonexistent. As most AMICS patients have the potential to improve LV function after revascularization, thus the criteria for use of MCS in AMICS patients should be less restrictive than the criteria used for identifying candidates for a heart transplant or durable LV assist devices. However, comorbidities, such as severe peripheral arterial disease, severe chronic pulmonary disease, advanced multiorgan failure, and especially hepatic failure, are associated with particularly poor outcomes [18,19], and the potential for any MCS device to improve outcomes in these patients is low. Even though recent data suggest that VA-ECMO in highly selected patients with refractory cardiac arrest and ongoing chest compression improves survival [20], the use of Impella in patients with a high likelihood of hypoxic brain injury (unwitnessed arrest, low-flow above >45 min, nonshockable primary recorded rhythm) cannot be generally encouraged. With the accuracy of currently available risk models, the treating shock team should make the decision of futility, and thus, future studies should focus on futility criteria as well as selection criteria.

## 6. Timing of Therapy

In experimental studies, the placement of Impella during coronary occlusion, even when prolonging the ischemia time, has been demonstrated to lower wall stress and reduce myocardial oxygen consumption, leading to a reduction in the infarct size compared with immediate revascularization [3,8]. Registry data suggest higher survival rates with Impella placement before revascularization than in patients where Impella is placed after PCI [21,22,23]. However, the level of evidence is low, as the data are subject to selection bias and other confounding factors. In patients with AMI and no shock, pre-PCI Impella has been shown to be safe in a feasibility trial [24], and the ongoing Door to Unload trial (ClinicalTrials.gov Identifier: NCT03947619) will provide insight into the potential benefit of pre-PCI Impella in AMI without shock. Until randomized data are available, the timing of Impella placement should be a balance between the severity of hemodynamic instability and the complexity of the coronary lesion, weighed against the fact that immediate revascularization is the only treatment demonstrated to improve outcomes in AMICS [25].

## 7. Evidence for Routine Use of Impella in AMICS

The evidence for the use of an axial flow pump in AMICS is low and based on two small randomized studies, conflicting retrospective case series and registry studies. The efficacy study of the LV Assist Device to Treat Patients With Cardiogenic Shock (ISAR-SHOCK) study was a feasibility study on 25 patients comparing the Impella 2.5 with the intra-aortic balloon pump (IABP) [26]. ISAR-SHOCK was designed to assess the effect on hemodynamic parameters which demonstrated increased cardiac output in the Impella group. In the IMPella versus IABP Reduces mortality in Stemi patients treated with primary PCI in severe cardiogenic shock (IMPRESS), 48 AMICS patients with refractory shock were randomized to Impella or IABP [27]. The study failed to identify a benefit of Impella CP. The study was based on the assumption that treatment with Impella would decrease the absolute 30-day mortality rate from 95% to 60%, which was not met, as mortality was 50% at 6 months in both groups. The majority of patients (>90%) had suffered OHCA and the leading cause of death was hypoxic brain injury. Schrage et al. performed a larger (*n* = 237) retrospective study with a propensity-matched comparison of patients fulfilling IABP-Shock 2 inclusion criteria but treated on a clinical basis with Impella 2.5 or CP vs patients from the IABP-Shock 2 trial [28]. Impella use was not associated with any improvement in 30-day all-cause mortality [28]. Further, in a recent retrospective study based on data from the CathPCI and Chest Pain-MI registries of the American College of Cardiology National Cardiovascular Data Registry, Dhruva et al. raised concerns about excess mortality in Impella compared with IABP-treated patients [29]. As there was no assessment of the hemometabolic state at the time of placement of the device, the study has a high risk of selection bias and uncontrolled bias. Opposed to these registry data, the National Cardiogenic Shock Initiative (NCSI) is a protocol-based approach to treating AMICS with mainly Impella CP. The protocol recommends early device placement (pre-PCI in 74%). With this approach, a survival rate of 72% at 30 days has been achieved [30] in a population with marked hemometabolic derangement at presentation (mean lactate 5.4 mmol/L). Although a numerically higher survival when compared with previously reported studies, direct comparison is difficult without a well-defined control group and longer follow-up.

Thus, there is currently no strong evidence to support the routine use of axial flow pumps in AMICS, and further evidence should be gained from adequately powered randomized trials. The Danish-German Cardiogenic Shock study (DanGer shock) is an ongoing trial randomizing ST-segment elevation AMI CS patients not presenting with OHCA and mandatory lactate >2.5 mmol/L [31]. The study aims at randomizing 360 patients to Impella CP or conventional management (IABP and VA-ECMO allowed). By the end of 2021, 75% of the sample size has been enrolled, and the results of the study are expected in 2023. In the United States, there is an ongoing initiative to initiate a randomized study (RECOVER IV) based on the NCSI algorithm with mandatory pre-PCI Impella placement. Enrollment in RECOVER IV is expected to start in 2022.

## 8. Escalation Strategies

**Figure 2:**
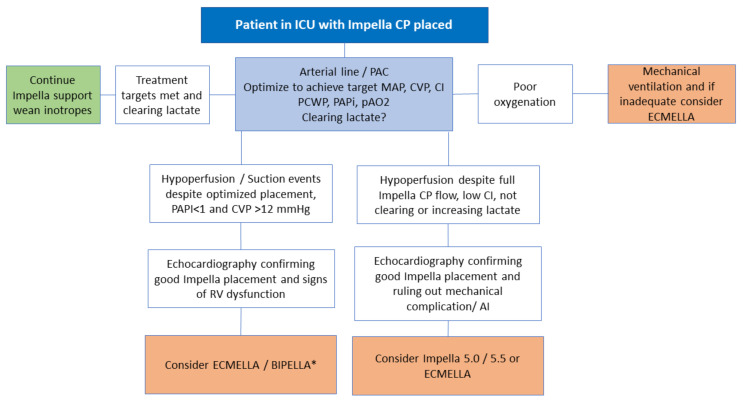
Decision flow chart on the identification of patients for a potential escalation of Impella CP in acute myocardial infarct-related cardiogenic shock. AI, aortic insufficiency; BIPELLA, Impella RP combined with left-sided Impella; ICU Intensive Care Unit; CI, cardiac index; CVP, central venous pressure; ECMELLA, VA-ECMO combined with Impella; MAP, mean arterial pressure; PAC, pulmonary artery catheter; pAO2 partial arterial pressure of oxygen; PCWP, pulmonary artery wedge pressure; PAPi, pulmonary artery pulsatility index; RV, right ventricular. * BIPELLA should only be considered in case of adequate oxygenation.

After placement of Impella CP in AMICS and revascularization, patients should be monitored for signs of inadequate circulatory support until recovery of native heart function. Escalation is usually done using more powerful Impella devices, such as Impella 5.0 and 5.5, a combination of Impella and VA-ECMO (ECMELLA), or in selected cases, Impella RP and CP in combination (BIPELLA), as shown in Figure 2. The combination of Impella and IABP should be avoided. A well-placed Impella CP in predominantly LV failure data from NCSI suggests that the need for escalation is relatively infrequent (9% of patients required escalation from Impella CP) [30]. To identify signs of inadequate circulatory support patient monitoring with an arterial line, a pulmonary artery catheter (to assess pulmonary artery pressure, central venous pressure, pulmonary artery pulsatility index, pulmonary capillary wedge pressure, cardiac output) together with serial measurements of lactate, and frequent echocardiography can provide high-quality monitoring [32]. The decision of escalation is complex and should not be based on a single hemodynamic measure but on the integration of several measures, and especially, the hemometabolic response to treatment is important (normalization of lactate). Ideally, inotropes should be weaned to lower myocardial oxygen consumption. 

## 9. Weaning

The possibility of weaning from the axial flow device in AMICS should be evaluated daily already from 24–48 h after imitating support. The evidence for the timing and the mode of weaning from an MCS device is very limited. In general, the patient must be stable with a pulsatile arterial waveform, on low-dose vasopressors, and with no or minimal inotropic support. Weaning should be guided by hemodynamic monitoring with a pulmonary artery catheter [32]. There are no validated echocardiographic cut-off values that predict successful weaning in Impella-supported patients.

## 10. Complications

The use of axial flow pumps in critically ill patients is associated with access site complications and complications related to the flow pump, that even with careful access site management and continuing assessment of placement cannot be avoided. The most frequent access site-related complication is bleeding complications, where the registry-based study by Dhruva et al. reported a 31% rate of major bleeding according to the Chest Pain-MI Registry. Of these, 11% were reported as access site bleeding, which was significantly higher than in the IABP-treated patients (3% access site-related) [29]. In the retrospective study by Schrage et al., moderate bleeding was seen in 19% of those treated with Impella, and severe life-threatening bleeding was in 10%, compared with 21% and 2%, respectively, in the IABP group [28]. In NCSI, access site bleeding requiring intervention occurred in 10% [30]. A recent Italian study reported 11% access site bleeding [33]. None of the studies reported the use of ultrasound-guided femoral arterial puncture, which should be done to reduce the risk of access site-related complications. In addition, there is a need for more uniform reporting of bleeding complications to better set treatment standards and compare data across studies. 

In a recent post hoc analysis, it has been demonstrated that a significant drop in hemoglobin in patients with acute coronary syndrome without overt bleeding was associated with mortality [34]. Less than 1% of patients had cardiogenic shock, and whether the same association to mortality is present in AMICS where the majority of deaths occur within hours of admission [35], is speculative. However, a drop in hemoglobin is very frequent in AMICS and may be more frequent with Impella treatment due to hemolysis. The importance of hemoglobin drop without overt bleeding should be addressed in future studies.

Unlike VA-ECMO, distal arterial protection is not readily feasible with Impella support and thus patients with existing peripheral arterial disease are at risk of limb ischemia. The reported risk of limb ischemia varies between 4% in NCSI and 12.6% in the IMP-IT registry [28,34]. 

Hemolysis is usually seen during the early course of management and is apparent as a rise in plasma-free hemoglobin, and in severe cases, a drop in hemoglobin. The risk of hemolysis increases dramatically with malposition of the devices and LV suction events. The presence of hemolysis should always prompt a reduction in P-levels until the problem is resolved, and should prompt immediate imaging to optimize the placement, and evaluate RV function, and volume status.

## 11. Conclusions

In a situation such as AMICS associated with critically low cardiac output, decreased myocardial contractility and increased wall stress, a treatment that unloads the myocardium and restores CO without increasing myocardial oxygen demand is theoretically appealing. Likely, the SCAI C patient with predominantly LV failure without prolonged cardiac arrest is the best candidate for axial flow pumps. Registry data suggest that pre-PCI Impella may be advantageous to post-PCI placement. However, several gaps in knowledge exist regarding optimal patient selection, futility criteria, timing, weaning, and escalation strategy, and until data from adequately sized randomized trials are available, immediate individual evaluation for MCS by a shock team is warranted when a patient is diagnosed with AMICS; see Table 1. 

## Figures and Tables

**Table 1:** Key Messages.

Experimental data suggest axial flow pumps may lower wall stress, reduce myocardial oxygen consumption and reduce infarct size during coronary occlusion.
Candidacy for MCS including Impella should be decided when a shock is diagnosed and decided by the shock team.
Impella CP is likely best suited in the SCAI class C patient with predominantly LV failure and objective signs of hypoperfusion (elevated lactate).
Registry data are conflicting and available randomized trials are not adequately powered for mortality. Until adequately sized randomized trials are available, the use of the device should be based on shock team evaluation.
Pre-PCI placement of Impella should be considered in hemodynamically compromised patients, especially those with complex coronary anatomy.
Patients should be monitored with a pulmonary artery catheter in the intensive care unit combined with frequent lactate measurements and imaging to screen for device displacement, biventricular failure, and a need for escalation.
Most frequent complications are accessing site-related bleeding and limb ischemia that are more frequent than what is seen in patients supported by IABP.

## Data Availability

Not applicable.
